# Impact of Persistent Iatrogenic Atrial Septal Defect following MitraClip

**DOI:** 10.3390/jcdd10010001

**Published:** 2022-12-22

**Authors:** Chieh-Ju Chao, Amith Seri, Bishoy Abraham, Juan M. Farina, Evelyn Fennelly, Megan Campany, Milagros Pereyra, Ebram F. Said, Courtney Kenyon, Ayman R. Fath, Sean Smith, Skye Buckner-Petty, Corbin A. Rayfield, David Fortuin, John P. Sweeney, Eric H. Yang, Chadi Ayoub, Mackram F. Eleid, Mohamad Alkhouli, Charanjit S. Rihal, David R. Holmes, Peter M. Pollak, Abdallah El Sabbagh, Jae K. Oh, Reza Arsanjani

**Affiliations:** 1 Department of Cardiovascular Diseases, Mayo Clinic Rochester, Rochester, MN 55902, USA; 2 Department of Cardiovascular Diseases, Mayo Clinic, Phoenix, AZ 85054, USA; 3 Department of Cardiovascular Diseases, Mayo Clinic, Jacksonville, FL 32224, USA

**Keywords:** mitral valve insufficiency, endovascular procedures, echocardiography

## Abstract

Background: Prior studies have reported conflicting results of persistent iatrogenic atrial septal defect (iASD) and its impact following a transcatheter edge-to-edge repair (TEER) procedure. This study aims to evaluate the incidence of iASD and its clinical and hemodynamic impact after a TEER. Methods: Consecutive patients who underwent a TEER procedure from June 2014 to September 2020 at the Mayo Clinic were identified. The presence of iASD was retrospectively identified on post-procedure transthoracic echocardiography (TTE) to group patients into an iASD+ group and an iASD− group for comparison of prognosis and cardiac function. Results: A total of 316 patients were included; the mean age was 79.1 ± 9.1 years, and 67.7% were male. Persistent iASD was diagnosed in 108 (34.2%) patients. There was no difference concerning all-cause mortality, heart failure hospitalization, and stroke/TIA between groups at follow-up (median follow-up of 9 months). Post-procedure TTE demonstrated no differences regarding right ventricle (RV) and left ventricle (LV) dimensions and function, including TAPSE (15.2 ± 4.6 vs. 15.4 ± 5.5 mm, *p* = 0.875), and LV ejection fraction (51.1 ± 14.0% vs. 51.3 ± 13.9%, *p* = 0.933). However, patients with iASD had higher RV systolic pressure (48.7 ± 14.4 vs. 45.5 ± 14.5 mmHg, *p* = 0.042) compared with patients without iASD. Conclusion: Notwithstanding higher RV pressures, patients with persistent iASD had no hemodynamic compromise and an equal prognosis compared with those without a residual atrial defect after a TEER procedure. These findings support the mid-term safety of procedures in which an interatrial defect needs to be created and would argue against the need for interventional closure.

## 1. Introduction

Transcatheter edge-to-edge mitral valve repair (TEER) has emerged as a treatment option for severe mitral regurgitation (MR) in patients with high surgical risk [1]. Part of the procedure requires atrial septum puncture to allow access to the left atrium, which causes iatrogenic atrial septal defect (iASD). It is reported that interatrial defects can persist after the procedure [2], with an incidence of persistent iASD after TEER ranging between 24–62% of cases [2,3,4,5]. 

Previous studies have evaluated the clinical, hemodynamic, and echocardiographic implications of persistent iASD after TEER, with divergent results [2,3,4,5]. While some evidence reported that patients with persistent iASD were not clinically compromised during follow-up, other studies showed that iASD may worsen right heart chambers hemodynamics and increase heart failure hospitalization (HFH) and mortality [2,3,4,5]. On the other hand, a study by Hoffmann and colleagues demonstrated an association between post-TEER iASD and improved hemodynamics due to volume and pressure relief of the left atrium [6]. Furthermore, a randomized single center study of patients with persistent iASD post-TEER comparing iASD interventional closure versus conservative therapy found no improvement in clinical outcomes at the 12-month follow-up [7,8]. However, the investigators noted a higher risk of HFH in patients with iASD independent of management strategy and recommended close follow-up in this group. 

Based on the studies mentioned above, the persistence of iASD after TEER procedures and its clinical relevance remain under debate. The aim of the current study was to investigate the clinical, functional, and hemodynamic impact of persistent iASD after TEER.

## 2. Materials and Methods

Consecutive adult patients (≥18 years old) who underwent a TEER procedure for severe MR between 1 June 2014 and 1 September 2020 were identified from the Mayo Clinic National Cardiovascular Diseases Registry database, which included patients from three major academic medical centers in Rochester, MN, Phoenix, AZ, and Jacksonville, FL. Post-procedure transthoracic echocardiography (TTE) was used to identify the presence of iASD; patients without a post-procedure TTE study were excluded. Patients were divided into two groups according to the presence of persistent iASD (iASD+, and iASD−). Baseline (pre-procedure) characteristics, follow-up clinical outcomes, and echocardiography data were collected.

Our primary endpoint was to compare clinical (mortality, HFH, stroke/TIA, NYHA functional class, and six-minute walk test) and echocardiography (right and left ventricle dimensions and function) outcomes between patients with persistent iASD+ and iASD− after TEER. A prespecified subgroup analysis was performed to compare (a) the effects of persistent iASD among patients with severe right ventricle (RV) systolic dysfunction at follow-up (defined as tricuspid annular plane systolic excursion (TAPSE) < 16 mm) [9], and (b) potential differences in outcomes according to the direction of the flow in cases of iASD+ (left to right direction vs. right to left or bilateral flow).

TTE was performed using standard ultrasound scanners (Philips iE33; Philips Medical Systems; GE Vivid E9, GE Healthcare) with a 3.5 MHz transducer, including postprocedure tissue Doppler imaging to determine the presence of persistent iASD. All measurements were performed as per American Society of Echocardiography (ASE) guidelines [10].

Descriptive analyses were conducted using frequencies (percentages) for categorical variables and means ± standard deviations (SD) or median (interquartile range) for continuous variables according to distribution. Categorical variables were compared using Fisher’s exact test or Chi-square test, while continuous variables were compared using the independent two-sample t-test or non-parametric tests. Cox regression was performed for survival analysis, and log rank test was used to compare survival difference between groups. All tests were two-tailed, and a *p*-value < 0.05 was considered statistically significant. 

Institutional review board (IRB) at Mayo Clinic evaluated and exempted this study, as conducted using retrospective data from established settings and involving established practices. IRB approved the waiver of informed consent as well, as the research project did not involve new patient care or interaction with patients; this project was entirely dependent on the use of previous imaging and chart data that was obtained during patients’ usual care.

## 3. Results

In total, 316 patients were included for the final analysis. The mean age was 79.1 ± 9.1 years, and 67.7% were males. The median follow-up period of the cohort was 9.0 months. During the follow-up period, 33 (10.4%) patients died. At post-procedure TTE, 108 (34.2%) had persistent iASD. There were no significant differences in baseline characteristics between patients with a persistent iASD and patients without persistent interatrial defects (Table 1).

**Figure 1:**
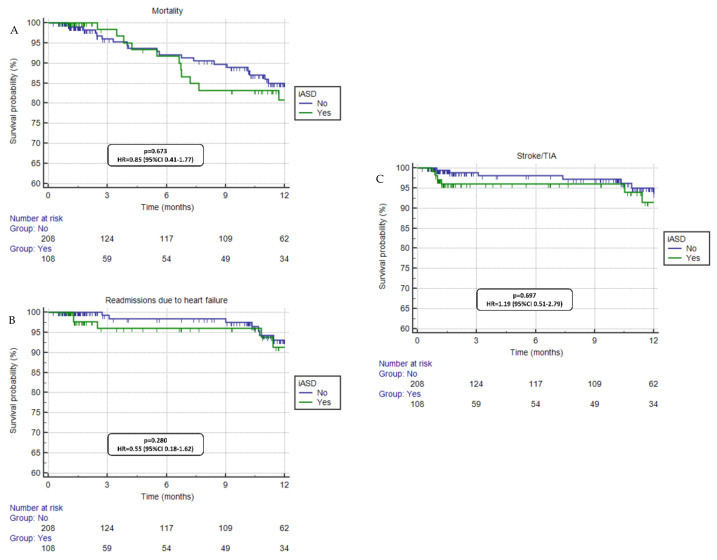
Clinical outcomes comparison between groups (iASD+ vs. iASD−). No significant differences were seen in mortality (panel **A**), readmissions due to heart failure (panel **B**) and stroke/TIA (panel **C**).

Following the procedure, overall mortality rate of the included population was 10.4%, 25.4% of patients were in advanced NYHA functional class (Class III-IV), 7.6% had a stroke/TIA, and 4.8% of patients had at least one HFH event. Regarding the clinical comparison between patients with iASD+ vs. iASD−, there were no differences between both groups in mortality rates (*p* = 0.673, HR = 0.85, 95%CI 0.41–1.77), stroke/TIA (*p* = 0.697, HR = 1.19, 95%CI 0.51–2.79), HFH events (*p* = 0.280, HR = 0.55, 95%CI 0.18–1.62), and NYHA functional class (*p* = 0.276) (Table 2, Figure 1). 

Post-procedure TTEs were performed at a median time of 9 months following the TEER. Regarding the comparison between patients with and without a persistent iASD, no differences were found in RV and LV dimensions, including RV end diastolic area (25.3 ± 6.9 vs. 29.9 ± 9.2 cm2, *p* = 0.073) and LV end diastolic volume (149.4 ± 69.6 vs. 142.3 ± 69.5 mm^3^, *p* = 0.482). Furthermore, no differences in RV systolic function were noted considering TAPSE (15.2 ± 4.6 vs. 15.4 ± 5.5 mm, *p* = 0.875). No significant differences were seen regarding LV systolic function, including LV ejection fraction (LVEF, 51.1 ± 14.0 vs. 51.3 ± 13.9 %, *p* = 0.933). In total, 7.2% of patients had residual severe MR, with no differences between both groups in the degree of residual MR (*p* = 0.779) and mitral valve diastolic mean gradient (4.6 ± 2.2 vs. 4.3 ± 2.1 mmHg, *p* = 0.289). The degree of tricuspid regurgitation (TR) was not different between the groups (*p* = 0.215) either. However, patients with iASD had higher RV systolic pressure (48.7 ± 14.4 vs. 45.5 ± 14.5 mmHg, *p* = 0.042) compared with patients without iASD (Table 3). 

**Figure 2:**
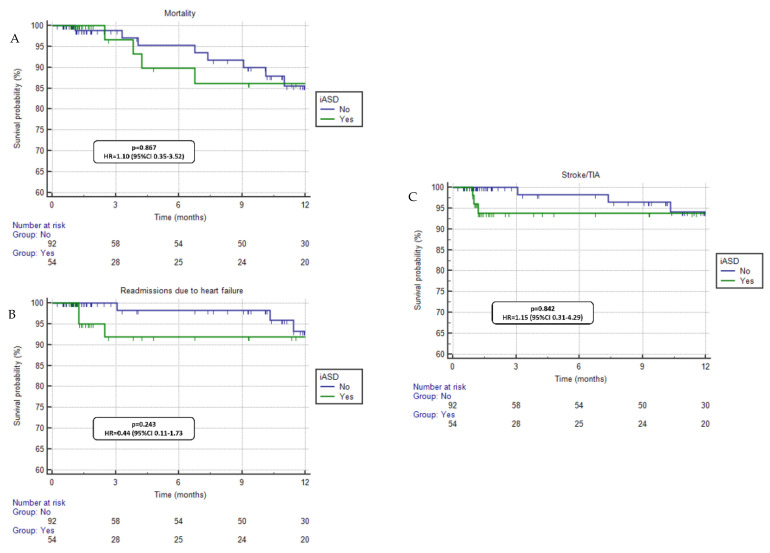
Clinical outcomes comparison between groups (iASD+ vs. iASD−) for patients with right ventricle systolic dysfunction. No significant differences were seen in mortality (panel **A**), readmissions due to heart failure (panel **B**) and stroke/TIA (panel **C**).

**Figure 3:**
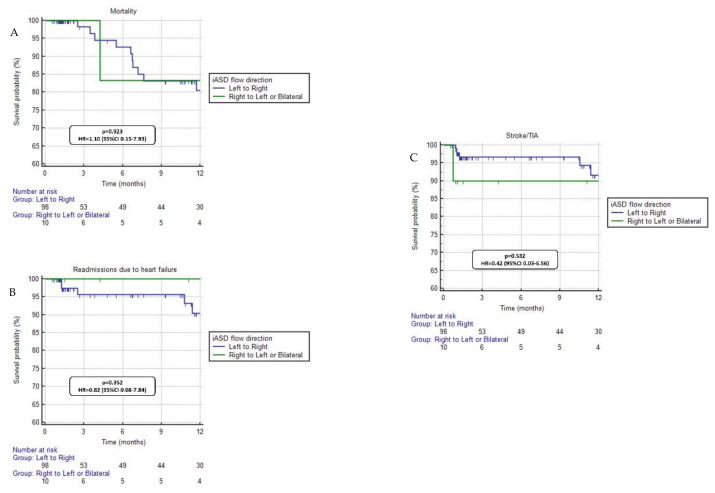
Clinical outcomes comparison for patients with a persistent interatrial septal defect according to the flow direction (left to right vs. right to left or bilateral). No significant differences were seen in mortality (panel **A**), readmissions due to heart failure (panel **B**) and stroke/TIA (panel **C**).

Regarding the subgroup analysis of patients with RV dysfunction (*n* = 146), no differences in mortality (*p* = 0.867, HR = 1.10, 95%CI 0.35–3.52), HFH (*p* = 0.243, HR = 0.44, 95%CI 0.11–1.73), and stroke/TIA (*p* = 0.842, HR = 1.15, 95%CI 0.31–4.29) were seen when comparing patients with and without a persistent iASD (Figure 2). In addition, when considering the analysis of the flow direction at the level of the interatrial defect in patients with a persistent iASD (*n* = 108), no prognostic differences were found between patients with left to right flow versus patients with right to left or bilateral flow regarding mortality (*p* = 0.923, HR = 1.10, 95%CI 0.15–7.93), HFH (*p* = 0.352, HR = 0.82, 95%CI 0.08–7.84), or stroke/TIA (*p* = 0.532, HR = 0.42, 95%CI 0.03–6.56) (Figure 3).

## 4. Discussion

In this retrospective study, we identified 34.2% of the patients who underwent a TEER procedure had a persistent iASD after the procedure. Our analysis demonstrated that the presence of a persistent iASD did not have a significant negative effect on clinical or hemodynamic outcomes when compared to patients without a persistent iASD.

Together with the spread of transcatheter structural interventions, trans-septal puncture during cardiovascular procedures have increased in recent times [11]. In particular, the MitraClip delivery system requires interatrial septum puncture and uses large bore catheters, resulting in iASD. The true incidence of persistent iASD after TEER has been estimated in previous studies with divergent results (the reported incidence has ranged between 24–62%) [2,3,4,5]. This wide reported range may be related to the diverse duration of follow-up and the diverse imaging tests used by previous investigations. Schueler et al. studied 66 patients who underwent MitraClip and completed 6 months follow-up; iASD was found in 50% of the patients using transesophageal echocardiography (TEE) [3]. A study of 53 patients by Alachkar et al. showed an incidence of persistent iASD of 62% using TEE at 6 months after the procedure [2]. Smith et al. reported a prevalence of 27% of iASD using TTE 6 months after the TEER [4]. Also using TTE, Toyama et al. detected an incidence of persistent iASD of 24% at a 1-year follow-up [5]. In our study, the incidence of persistent iASD was 34.2%, which is similar to the prior studies which used TTE to detect this post-procedural defect and supports the idea that persistent iASD is a common finding following a TEER procedure.

Prior studies have demonstrated the controversial impact of persistent iASD on clinical, hemodynamic, and echocardiographic outcomes after a TEER procedure [2,3,4,5]. Toyama et al. reported that the presence of a persistent iASD was associated with right-sided heart chambers enlargement, worse TR, and a higher HFH rate [5]. Likewise, Schueler et al. demonstrated that persistent iASD was associated with worse clinical outcomes and increased mortality at a 6-month follow-up [3]. On the other hand, Alachkar et al. reported that, despite an increase in RV diameter in patients with persistent iASD, these patients were not clinically compromised compared to patients without persistent iASD [2]. Additionally, in a small prospective study evaluating 28 patients only immediately after the MitraClip procedure, Hoffmann et al. demonstrated that iASD may have positive hemodynamic effects, resulting in a drop in left atrial pressure and thereby resulting in a LV preload reduction [6]. Our results reinforce the idea that a persistent iASD is not associated with worse clinical and hemodynamic outcomes concerning all-cause mortality, HFH, cerebrovascular events, and NYHA functional class. Even though patients with persistent iASD had a slight but significantly higher RV systolic pressure in our cohort, this was not accompanied with deterioration in the RV or LV systolic function. These findings support the mid-term safety of procedures in which an interatrial defect needs to be created and these results imply that interventional closure of iASD may not be necessary in certain cases. 

In the same way, the benefits of iASD closure post TEER are controversial. One study reported that iASD closure after TEER led to a volume shift from RV to LV with improvement in heart failure symptoms at 1-month [12]. However, in a single center randomized study by Lurz et al., interventional closure of a relevant iASD did not result in improved clinical outcomes at 12 months post-TEER when compared with conservative therapy [7,8]. It has been proposed that the negative effects of iASD on right-sided volume overload might be counterbalanced by the positive implications of volume and pressure relief to the left atria. Indeed, in a recent trial the creation of an iASD reduced left ventricular pressures in patients with heart failure with a preserved ejection fraction [13]. 

The analysis of the subgroup of patients with severe RV systolic dysfunction was performed with the hypothesis that patients with more compromised RV function could not tolerate the presence of iASD due to the potential volume increase in the right heart chambers. However, no prognostic differences were seen between patients according to the presence of an iASD in this subgroup (Figure 2). As the RV adaptation to diseases is determined not only by the presence of volume/pressure overload but also by the degree of the overload [14] (among other factors), the degree of volume overload generated by the residual iASD might not have been sufficient to generate a clinical impact. 

The persistence of an iASD can lead to left to right shunts and occasionally to right to left or bilateral shunts. In a previous publication by Morikawa et al., 5% of the studied patients had right to left shunt. In that study the presence of right to left shunt was attributed to a more severe compromise of right heart chambers and was associated with major adverse cardiovascular events at follow-up [15]. In our cohort, we did not find clinical or prognostic differences in patients with iASD according to the direction of the flow (Figure 3). However, only a small proportion of our patients presented right to left or bilateral shunts, which could have limited the results of this specific comparison. Nonetheless, the careful assessment of hemodynamics before the TEER procedure and the accurate distinction between different types of shunts when assessing the clinical significance of iASD after TEER seems of relevance. 

Our research, to the best of our knowledge, included the largest cohort of patients to date with regard to this particular topic. We used TTE to diagnose iASD at 9 months follow-up, when some previous studies used TEE and a different follow-up period. Whether the use of TEE could have allowed us to detect more cases of post-procedure iASD remains unknown; nevertheless, TTE still remains the preferred initial diagnostic modality for the detection and diagnosis of ASD [16].

Our conclusions may be limited by the retrospective nature of the study. Patients had different follow-up periods and some loss of follow-up data existed, more precisely regarding readmissions after the procedure, considering that our centers are destination hospitals and often patients performed their follow-up at different facilities. A complete comparison between baseline and postoperative echocardiography characteristics could have helped to strengthen our results, however the lack of preoperative data did not allow a thorough comparison.

## 5. Conclusions

Our study showed that the presence of a persistent iASD post-TEER did not lead to increased adverse events or worse cardiac function. Notwithstanding higher RV pressures, patients with persistent iASD did not have hemodynamic compromise based on TTE evaluation. These findings support the mid-term safety of procedures in which an interatrial defect needs to be created and implies that interventional closure of iASD may not be necessary in certain cases.

## Figures and Tables

**Table 1:** Baseline characteristics of the included population.

	Iatrogenic ASD Present (*n* = 108)	Iatrogenic ASD Not Present (*n* = 208)	Total (*n* = 316)	*p* Value
Age, years	77.9 ± 9.9	79.7 ± 8.5	79.1 ± 9.1	0.101
Sex, *n* (%)				0.972
Female	35 (32.4%)	67 (32.2%)	102 (32.3%)	
Male	73 (67.6%)	141 (67.8%)	214 (67.7%)	
Height, cm	169.9 ± 10.7	168.6 ± 9.9	169.0 ± 10.2	0.245
Weight, Kg	77.6 ± 19.5	78.7 ± 19.1	78.4 ± 19.2	0.613
Body Mass Index, Kg/m2	26.6 ± 5.1	27.5 ± 5.4	27.2 ± 5.3	0.136
Smoking, *n* (%)	4 (3.7%)	4 (1.9%)	8 (2.5%)	0.339
Hypertension, *n* (%)	94 (87.0%)	182 (87.5%)	276 (87.3%)	0.907
Diabetes, *n* (%)	26 (24.1%)	51 (24.6%)	77 (24.4%)	0.912
Prior Myocardial infarction, *n* (%)	33 (30.6%)	58 (27.9%)	91 (28.9%)	0.583
Prior PCI, *n* (%)	25 (23.1%)	61 (29.3%)	86 (27.2%)	0.242
Prior CABG, *n* (%)	35 (32.4%)	62 (29.8%)	97 (30.7%)	0.635
STS Risk (for isolated mitral valve repair)	7.5 ± 5.2	7.6 ± 5.7	7.5 ± 5.5	0.939
Pacemaker, *n* (%)	14 (13.0%)	34 (16.3%)	48 (15.2%)	0.427
Prior Stroke, *n* (%)	12 (11.1%)	23 (11.1%)	35 (11.1%)	0.989
Prior Peripheral artery disease, *n* (%)	32 (29.6%)	53 (25.5%)	85 (26.9%)	0.430
End stage kidney disease, *n* (%)	3 (2.8%)	7 (3.4%)	10 (3.2%)	0.777
Atrial Fibrillation, *n* (%)	77 (71.3%)	134 (64.4%)	211 (66.8%)	0.219
Six-minute walk distance, m	326.4 ± 110.9	299.9 ± 107.6	308.1 ± 109.1	0.101
NYHA FC, *n* (%)				0.923
Class I	1 (0.9%)	4 (1.9%)	5 (1.6%)	
Class II	22 (20.4%)	39 (18.8%)	61 (19.3%)	
Class III	67 (62.0%)	128 (61.5%)	195 (61.7%)	
Class IV	18 (16.7%)	37 (17.8%)	55 (17.4%)	
NTproBNP, pg/ml	2198.0 (4958.0)	1958.0 (3409.0)	2020.0 (4147.5)	0.162
Degenerative Mitral valve disease, *n* (%)	92 (85.2%)	177 (85.1%)	269 (85.1%)	0.983

PCI: percutaneous coronary intervention. CABG: coronary artery bypass graft.

**Table 2:** Clinical follow-up observations.

	Iatrogenic ASD Present (*n* = 108)	Iatrogenic ASD Not Present (*n* = 208)	*p* Value
Six-Minute Walk Test, m	322.2 ± 131.9	305.0 ± 121.1	0.397
NYHA FC, *n* (%)			0.276
Class I	30 (40.0%)	60 (39.0%)	
Class II	31 (41.3%)	50 (32.5%)	
Class III	12 (16.0%)	36 (23.4%)	
Class IV	2 (2.7%)	8 (5.2%)	
Readmission due to Heart Failure, *n* (%)	7 (6.5%)	8 (3.9%)	0.280
Stroke/TIA	7 (6.5%)	17 (8.2%)	0.697
Mortality, *n* (%)	12 (11.1%)	21 (10.1%)	0.673

**Table 3:** Transthoracic echocardiography outcomes at follow-up.

	Iatrogenic ASD Present	Iatrogenic ASD Not Present	*p*-Value
MV mean gradient (mmHg)	4.6 ± 2.2	4.3 ± 2.1	0.289
LVEF (%)	51.1 ± 14.0	51.3 ± 13.9	0.933
LVEDV (mL)	149.4 ± 69.6	142.3 ± 69.5	0.482
LVESV (mL)	82.7 ± 56.1	76.0 ± 56.1	0.410
RVEDA (cm^2^)	25.3 ± 6.9	29.9 ± 9.2	0.073
RVSP (mmHg)	48.7 ± 14.4	45.5 ± 14.5	0.042
TAPSE (mm)	15.2 ± 4.6	15.4 ± 5.5	0.875
Mitral valve regurgitation (%)			0.779
Trivial	7.1	4.6	
Mild	44.4	41.8	
Moderate	37.4	48.5	
Severe	11.1	5.2	
Tricuspid valve regurgitation (%)			0.215
Trivial	15.6	15.7	
Mild	40.6	47.6	
Moderate	26.0	25.7	
Severe	17.7	11.0	

MV: mitral valve; LVEF: left ventricle ejection fraction; LVEDV: left ventricle end diastolic volume; LVESV: left ventricle end systolic volume; LV: left ventricle; RVSP: right ventricle systolic pressure; RV: right ventricle; RVEDA: right ventricle end diastolic area; TAPSE: tricuspid annular plane systolic excursion; LA: left atrium.

## Data Availability

The data presented in this study are available on request from the corresponding author.
